# Target mismatch criteria in acute ischemic stroke patients with distal-medium vessel occlusion

**DOI:** 10.1093/esj/23969873251362205

**Published:** 2026-01-01

**Authors:** Giorgio Busto, Andrea Morotti, Ilaria Casetta, Francesco Arba, Guido Fanfani, Francesco Impagliazzo, Francesco Loverre, Andrea Ginestroni, Umberto Pensato, Alessandro Padovani, Enrico Fainardi

**Affiliations:** Neuroradiology Unit, Department of Radiology, Careggi University Hospital, Florence, Italy; Neurology Unit, Department of Clinical and Experimental Sciences, University of Brescia, Brescia, Italy; IRCCS San Camillo Hospital, Venice, Italy; Stroke Unit, Careggi University Hospital, Florence, Italy; Neuroradiology Unit, Department of Radiology, Careggi University Hospital, Florence, Italy; Neuroradiology Unit, Department of Radiology, Careggi University Hospital, Florence, Italy; Neuroradiology Unit, Department of Radiology, Careggi University Hospital, Florence, Italy; Neuroradiology Unit, Department of Radiology, Careggi University Hospital, Florence, Italy; Department of Biomedical Sciences, Humanitas University, Milan, Italy; Department of Neurology, IRCCS Humanitas Research Hospital, Rozzano, Milan, Italy; Neurology Unit, Department of Clinical and Experimental Sciences, University of Brescia, Brescia, Italy; Neuroradiology Unit, Department of Experimental and Clinical Biomedical Sciences, University of Florence, Florence, Italy

**Keywords:** Acute ischemic stroke, distal medium vessel occlusion, CT-perfusion, target mismatch criteria, infarct core volume, endovascular treatment%

## Abstract

**Introduction:**

The efficacy of endovascular treatment (EVT) in ischemic stroke patients with distal-medium vessel occlusion (DMVO) remains unclear. We evaluated whether CT-perfusion target mismatch criteria (TMC) could predict functional independence in patients with M2 non- or codominant middle cerebral artery DMVO.

**Materials and methods:**

This retrospective study analyzed consecutive patients with M2 DMVO receiving EVT and imaged with multimodal CT study protocol within 24 h from onset. A receiver operating characteristic curve analysis was used to identify the infarct core volume cutoff to predict functional independence (modified Rankin Scale 0–2 at 3-months). This parameter was subsequently considered as part of TMC together with penumbra volume ⩾ 10 mL and mismatch ratio ⩾1.2. The association between TMC and functional independence was tested with logistic regression.

**Results:**

A total of 115 patients with M2 were included. Infarct core volume had good discriminative ability for functional independence (AUC 0.75; 95%CI 0.64–0.84) and the best cut-off value was ⩽30 mL (77% sensitivity, 61% specificity, 69% positive predictive value, 70% negative predictive value). TMC were independently associated with functional independence (OR [odds ratio] = 6.50, 95%CI = 2.37–17.77, *p* < 0.001), excellent outcome (modified Rankin scale 0–1 at 3-months, OR = 3.28, 95%CI = 1.30–8.31, *p* = 0.012) and final infarct volume (*B* = −35.52, *p* = 0.004). After including interaction terms, a significant treatment effect on functional independence was observed between successful recanalization and TMC (OR = 3.82, 95%CI = 1.64–8.89, *p* = 0.002).

**Discussion and conclusion:**

In patients with M2 non- or codominant DMVO receiving EVT, TMC identified as core volume ⩽30 mL, penumbra volume ⩾ 10 mL, and mismatch ratio ⩾ 1.2, were associated with better functional outcome. Our findings suggest that functional independence after EVT was not directly related to successful recanalization, which is indeed effective only in patients with a favorable baseline imaging profile, including a small infarct core size, and in the presence of small penumbra volumes.

## Graphical Abstract

**Graphical abstract:**
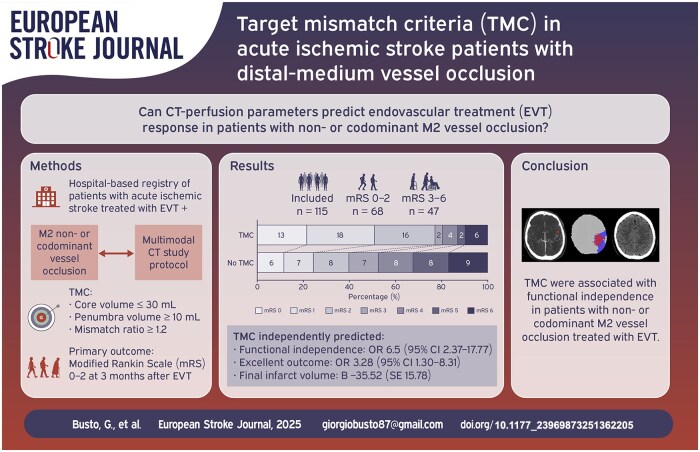

## Introduction

The efficacy of endovascular treatment (EVT) for acute ischemic stroke (AIS) patients suffering from large vessel occlusion (LVO) within 24 h from symptoms onset is well established.^1^ However, whether EVT is the optimal treatment option for AIS patients with distal-medium vessel occlusion (DMVO) remains questionable.^2^ Several studies demonstrated that patients with middle cerebral artery (MCA)-M2 vessel occlusion, which represents the largest proportion of DMVO cases, could effectively benefit from EVT.^3,4^ These patients achieved better clinical outcomes and similar successful reperfusion rates when compared with those with MCA-M1 vessel occlusion.^5,6^ However, more recently, two randomized clinical trials (RCTs), namely ESCAPE-MeVO (endovascular treatment of stroke due to medium-vessel occlusion)^7^ and DISTAL (endovascular treatment of stroke due to occlusion of medium or distal vessels),^8^ predominantly enrolling patients with M2 vessel occlusion (50.0% and 44.0%, respectively), showed that patients with DMVO undergoing EVT failed to achieve better functional outcomes compared with those managed with best medical therapy. Therefore, it remains unclear whether a subgroup of patients with DMVO might actually benefit from EVT.^9^ Perfusion imaging has been successfully adopted in AIS patients with LVO to assess eligibility for reperfusion therapies up to 24 h from symptoms onset, evaluating a combination of parameters collectively called target mismatch criteria and represented by infarct core and penumbra volumes and mismatch ratio (hypoperfused divided by infarct core volumes).^10^ In patients with M2 vessel occlusion undergoing EVT, penumbra volumes of target mismatch (penumbra volume > 15 mL with mismatch ratio > 1.8 and penumbra volume > 10 mL with mismatch ratio > 1.2, respectively) were previously associated with good clinical outcome.^11^ In contrast, how the CT perfusion-estimated baseline ischemic core volume influences the treatment effect of EVT on outcomes in DMVO patients remains less clear. While one study found an association between recanalization and good functional outcomes in patients with core volume up to 40 mL,^12^ another study found no association between ischemic core volume and outcome in this population.^13^ Therefore, the combination and relative proportion between ischemic core and penumbra might be more informative, compared to their individual assessment, to predict the functional outcome and treatment effect of EVT in DMVO patients. Thus, the aim of our study was two-fold: (i) to identify the optimal infarct core volume threshold for outcome prediction in patients with M2 vessel occlusion receiving EVT up to 24 h after stroke onset; (ii) to evaluate whether the target mismatch criteria, based on this newly identified core volume cut-off and previously accepted parameters of target mismatch defining penumbral profile, could predict functional independence in this subgroup of DMVO patients.

## Methods

### Data availability, standard protocol approval, and patient consent

The data analyzed in this study will be available and shared by the corresponding Author upon reasonable request from any qualified researcher for the purpose of replicating the results after clearance by the local ethics committee. This cohort study was approved by the local ethics boards and clinical information was recorded during routine clinical activity (PN 26299). Written informed consent was obtained from each patient or their legally authorized representatives at admission.

### Patient selection

**Figure 1.**
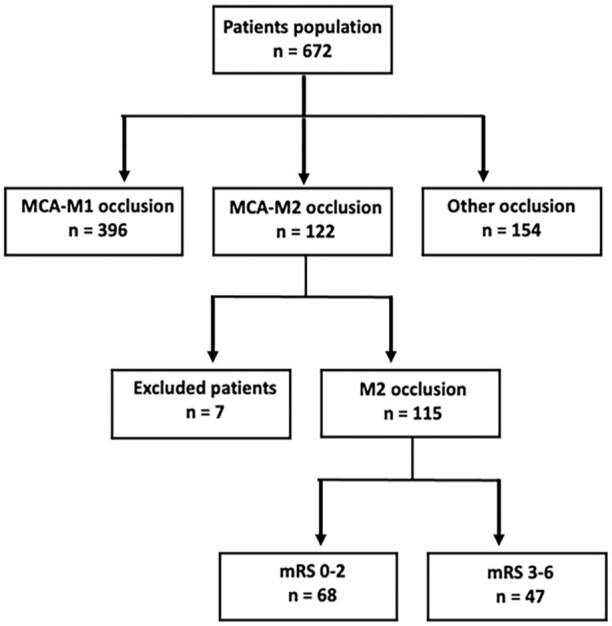
Flowchart of study population selection. MCA indicates middle cerebral artery, mRS modified Rankin-Scale.

In this observational retrospective study, we analyzed a prospectively collected cohort of 115 consecutive AIS patients with M2-MCA non- or codominant DMVO treated with EVT and admitted to our Hospital between January 2017 and December 2023. All patients underwent non-contrast computed tomography (NCCT), multiphase CT-angiography (mCTA) of the cervical and intracranial vessels and CT-perfusion (CTP) at admission within 24 h of symptom onset. Patients were included if they presented to the emergency department with the following criteria: (1) diagnosis of AIS within 24 h from witnessed symptom onset or time last seen well; (2) evidence of M2-MCA segment occlusion on CTA; (3) selected for receiving EVT; (4) CTP performed at admission and (5) undergoing follow-up NCCT imaging performed at 24 ± 12 h. Exclusion criteria were: (1) age <18 years; (2) pregnancy; (3) severe pre-stroke disability defined as modified Rankin Scale (mRS) ⩾3; (4) detection of intracerebral hemorrhage on admission NCCT; (5) contraindications to iodinated contrast agent; (6) poor quality of CT acquisition due to motion artifacts; (7) inability to complete the multi-modal CT protocol or the conventional digital subtraction angiography (DSA) study at baseline and/or 24-h follow-up NCCT. The M2 segment was defined as the vessel patenting from the genu of the main trunks of the MCA and encompassing these and their successive branches, on a vertical plane toward the most distal part of the insular circular sulcus. A distal M2 vessel occlusion (nearby or above the mid-height of the insula) was anatomically identified as non- or codominant M2 branch occlusion, in line with a previous study.^12^ Multivessel occlusions were not included in this study. The definition and identification of MCA occlusions was assessed by an experienced neuroradiologist (more than 10 years of experience). The study selection process is shown in Figure 1.

### Clinical assessment

Clinical, demographic and technical data were collected from the patient’s medical records and a prospectively maintained institutional stroke database by trained investigators blinded to the outcomes of interest, including age, sex, pre-stroke functional status (mRS), the presence of stroke risk factors, the interval between symptom onset and neuroimaging, the initial National Institute of Health Stroke Scale (NIHSS) score, the use of intravenous thrombolysis (IVT) and EVT.

### Imaging acquisition

All imaging protocols were conducted on a 128-slice scanner (Philips Brilliance iCT, Best, the Netherlands). NCCT helical scans were performed from the skull base to the vertex using these following imaging parameters: 120 kV, 340 mA, 0.6 collimation, 1 s/rotation, and table speed of 15 mm/rotation. CTA of the cervical and intracranial vessels was performed as follows: 0.7 mL/kg contrast (maximum 90 mL), 5- to 10-s delay from injection to scanning, 120 kV, 251 mAs, 0.75 s/rotation, 0.8/0.4 mm thick slices (imbricated slices), scan time 4 s. CTA covered from the carotid bifurcation to vertex. The second and third phases were acquired after a delay of 4 s that allows for table repositioning to the skull base. Scanning duration for each additional phase was 3.4 s. The axial images were reconstructed at 0.4 mm overlapping sections, and MIP multiplanar reconstructions for axial, coronal, and sagittal images of the circle of Willis were performed with 10 mm thickness at 3 mm intervals. CTP studies were obtained with a dynamic first-pass bolus-tracking methodology according to a two-phase imaging protocol, to avoid the truncation of time density curves, with a toggling table technique. The two-phase acquisition consisted of a first phase every 3.2 s for 60 s and an additional second phase every 15 s for 113 s, which started 5 s after the automatic injection of 40 mL of non-ionic contrast agent, followed by a saline flush of 40 mL at the rate of 4 mL/s. Sections of 8 cm length (across the *z*-axis) were acquired at 5 mm slice thickness. The other acquisition parameters were 80 kV, 150 mAs, and 0.33 rotation time. All CTP source images were reconstructed with a standard filter and display field of view (DFOV) of 25 cm.

### Imaging processing and analysis

The extent of early ischemic changes was evaluated on baseline NCCT using the ASPECTS methodology.^14^ mCTA was graded by two diagnostic neuroradiologists, (with more than 10 years and more than 20 years of experience, respectively, Cohen *K* > 0.8), both blinded to clinical information. According to a previously published scoring system, arterial collateral supply was graded on a 6-point scale. Grades 0–3 were classified as poor and grades 4–5 as good collaterals.^15^ CTP study was processed by commercially available delay-insensitive deconvolution automated software (Olea Sphere Version 3.0 SP23; Olea Medical, La Ciotat, France), using standard singular value decomposition method according to manufacturer instructions. All steps were checked for errors, including motion correction, smoothing, and evaluation of time density curves and selection of arterial input and venous output functions. As recommended by the vendor, total hypoperfused tissue and ischemic core volumes were defined as ischemic brain regions with time-to-maximum (Tmax) threshold values >6 s (Tmax >6 s) and relative cerebral blood flow (rCBF) threshold values less than 40% of normally perfused tissue (rCBF < 40%), respectively. The difference between the extent of Tmax >6-s and rCBF < 40% volumes was considered as ischemic penumbra. Mismatch ratio was defined as the Tmax >6-s volumes divided by rCBF <40% volumes. All these parameters were automatically segmented and calculated by the software. According to a previous publication,^16^ penumbra volume ⩾10 mL and mismatch ratio ⩾1.2 were considered as favorable penumbra profile. The recanalization rate was assessed on DSA at the end of the EVT procedure using the modified treatment in cerebral ischemia (mTICI). Successful recanalization was defined as mTICI score of 2b-3.^17^ Hemorrhagic transformation (HT) was classified on NCCT at 24 h from symptom onset/last known well according to the European Cooperative Acute Stroke Study (ECASS)-II criteria.^18^ Symptomatic intracranial hemorrhage (sICH) was considered as any intracranial hemorrhage associated with a ⩾4-point increase in NIHSS.^19^ Final infarct volumes were calculated on follow-up NCCT at 24 h after symptom onset/last known well with a semi-automated computer-assisted planimetric measurement with ITK-SNAP 3.8.0 software.

### Outcomes

The primary outcome was functional independence defined as a modified Rankin Scale (mRS) score of 0–2 at 3-months, which was evaluated by a stroke physician or a trained and certified neurology study nurse. All mRS raters were blinded to imaging and clinical information. Secondary outcomes included excellent functional outcome defined as mRS 0–1 at 3-months and final infarct volume, defined as the total lesion demarcation in follow-up CT 24 h after admission. Safety outcomes were HT and sICH, respectively.

### Statistical analysis

Continuous variables were summarized as median (interquartile range (IQR)) or mean (standard deviation (SD)) as appropriate based on their distribution assessed with the Shapiro-Wilk test. Mann-Whitney test and Student’s *t* test were used to compare continuous variables with non-normal and normal distributions, respectively. Categorical variables were summarized as count (percentage) and compared using the chi-square test. Baseline clinical and radiological characteristics were reported with descriptive statistics and compared between patients who achieved primary outcome versus those who did not. The discriminative ability of different infarct core volumes for identifying patients with good functional outcome (mRS 0–2 at 3-months) was analyzed using area under the curve (AUC) Receiver Operating Characteristic (ROC) curves and optimal sensitivity, specificity, positive predictive value, and negative predictive values. The optimal discriminative cut-off was identified using the Youden Index. Variables associated with good functional outcome were assessed using multivariable logistic regression, adjusting for age, admission NIHSS, mRS pre-stroke, collateral score, reperfusion status, and any variable showing significance at *p* < 0.1 in univariable analysis. The aforementioned multivariable logistic model was also adopted for predicting excellent functional outcome (mRS 0–1), HT and sICH. Variables associated with final infarct volume at 24 h were assessed using multivariable linear regression adjusting for age, admission NIHSS, mRS pre-stroke, collateral score, reperfusion status, and any variable showing significance at *p* < 0.1 in univariable analysis. Multicollinearity among covariates in the fully adjusted models was assessed using the generalized variance inflation factor (GVIF), with a threshold value of 5 adopted as the criterion to indicate potential collinearity. Backward elimination was used in all models to reach a final parsimonious model that avoids overfitting. All analyses were performed with the statistical packages SPSS version 25.0 (www.spss.com) and MedCalc (www.medcalc.org). Statistical significance was set at two-sided *p* < 0.05.

## Results

### Patient Characteristics

We screened 122 potentially eligible patients with AIS suffering from DMVO, 7 of whom were excluded due to poor CT acquisition quality related to motion artifacts (*n* = 3) and pre-stroke mRS ⩾3 (*n* = 4). Overall, 115 patients were included in the study (Figure 1). Table 1 summarizes patient characteristics and shows the comparison between subjects with (*n* = 68; 59.1%) and without (*n* = 47; 40.9%) functional independence at 3-months. Following univariable logistic regression analysis, DMVO patients with unfavorable outcome were older (*p* < 0.001) and predominantly women (*p* = 0.004) compared with those with good outcome and showed a significant higher pre-stroke disability and NIHSS at admission (*p* < 0.001). Patients achieving functional independence had better collaterals (*p* = 0.009) and smaller infarct core volume (*p* = 0.004) than subjects with poor outcome. Patients with good outcome had higher successful recanalization rate (*p* = 0.016), NIHSS at 24 h (*p* < 0.001) and smaller final infarct volume (*p* = 0.004), than those without functional independence.

**Table 1.** Study population characteristics according to functional outcome.

Variable	mRS 0–2	mRS 3–6	*p* Value
	*n* = 68	*n* = 47	*n* = 115
Age, mean (±SD), years	71.0 (13.6)	79.9 (10.2)	<0.001
Sex, woman, *n* (%)	32 (47.0)	35 (74.4)	0.004
mRS before stroke, median (IQR)	0 (0–1)	0 (0–2)	<0.001
Admission NIHSS, median (IQR)	10 (8–17)	17 (11–22)	<0.001
ASPECTS, median (IQR)	8 (8–9)	8 (7–9)	0.140
tPA before EVT, *n* (%)	34 (47.2)	17 (36.1)	0.194
Onset-to-CT time, min, median (IQR)	253 (190–444)	275 (180–450)	0.800
Good arterial collaterals, *n* (%)	55 (80.8)	29 (61.7)	0.009
rCBF < 40% infarct core volume, median (IQR)	14.8 (6.8–29.4)	31.2 (10.4–35.6)	0.004
Tmax>6-seconds volume in mL, median (IQR)	50.4 (34.1–84.6)	58.0 (29.4–85.5)	0.302
Mismatch volume in mL, median (IQR)	33.3 (17.3–57.3)	22.7 (12.8–46.1)	0.209
Mismatch ratio ⩾1.2	60 (88.2)	40 (85.1)	0.303
Onset-to-reperfusion time, min, median (IQR)	363 (280–553)	400 (300–560)	0.087
mTICI score 2b-3, *n* (%)	63 (92.6)	34 (72.3)	0.016
Hemorrhagic transformation, *n* (%)	16 (22.2)	10 (21.2)	0.856
sICH, *n* (%)	2 (2.7)	4 (8.5)	0.170
NIHSS at 24 h, median (IQR)	5 (2–11)	17 (11–22)	<0.001
Infarct volume at 24 h in mL, median (IQR)	16.0 (5.3–30.1)	28.8 (17.3–66.5)	0.004

SD: standard deviation; mRS: modified Rankin Scale; IQR: interquartile range; NIHSS: National Institutes Health Stroke Scale; ASPECTS: Alberta Stroke Program Early CT Score; tPA: tissue plasminogen activator; EVT: endovascular treatment; NCCT: non-contrast computed tomography; MCA: middle cerebral artery; ICA: internal carotid artery; rCBF: relative cerebral blood flow; Tmax: time to maximum concentration; mTICI: modified treatment in cerebral infarction score; sICH: symptomatic intracerebral hemorrhage.

### Prognostic performance of ischemic core volume

**Figure 2.**
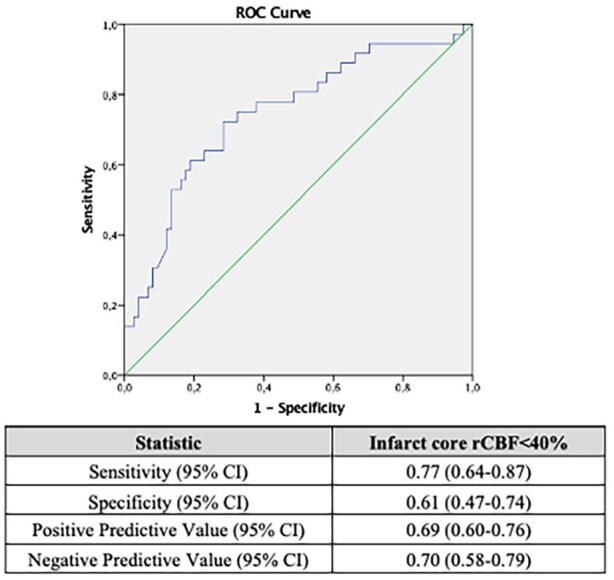
Infarct core volume optimal value for recognizing DMVO patients with good functional outcome as calculated using ROC curves. A rCBF<40% threshold of ⩽30 mL was found as the best cut-off. DMVO: distal-medium vessel occlusion; ROC: Receiver Operating Characteristic; rCBF: relative cerebral blood flow; CI: confidence interval; Outcome of interest: modified Rankin Scale 0-2 at 3-months.

ROC curve analysis, as shown in Figure 2, demonstrated that infarct core volume had good discriminative ability for favorable clinical outcome (AUC 0.75; 95%CI 0.64–0.84). The best cut-off value for predicting functional independence was ⩽30 mL (77% sensitivity, 61% specificity, 69% positive predictive value, 70% negative predictive value). Table 2 reports sensitivity, specificity, positive predictive value, and negative predictive value across different baseline infarct core cut-offs: 10-20-30-40-50 mL. Of note, rCBF < 40% infarct core volume ⩽30 mL was more frequent (*p* < 0.001) in patients with good than in subjects with poor outcomes (52/68; 76.4% vs 21/47; 44.6%).

**Table 2.** Predictive values of different CT-perfusion infarct core volumes for functional independence.

Infarct core rCBF < 40%	Sensitivity (95%CI)	Specificity (95%CI)	Positive predictive value (95%CI)	Negative predictive value (95%CI)
⩽10 mL	0.68 (0.51–0.81)	0.46 (0.35–0.58)	0.41 (0.34–0.48)	0.72 (0.61–0.81)
⩽20 mL	0.69 (0.57–0.80)	0.56 (0.41–0.71)	0.68 (0.62–0.77)	0.55 (0.44–0.65)
**⩽30 mL**	**0.77 (0.64–0.87)**	**0.61 (0.47–0.74)**	**0.69 (0.60–0.76)**	**0.70 (0.58–0.79)**
⩽40 mL	0.63 (0.52–0.77)	0.57 (0.34–0.78)	0.86 (0.79–0.91)	0.25 (0.17–0.35)
⩽50 mL	0.61 (0.50–0.70)	0.60 (0.26–0.87)	0.94 (0.88–0.97)	0.12 (0.7–0.20)

rCBF: relative cerebral blood flow.

In bold, Youden index cut-off point.

### Association between target mismatch criteria and outcomes

**Figure 3.**
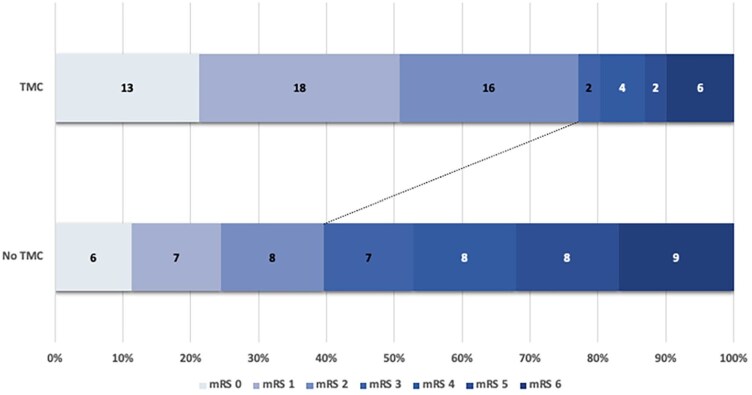
Distribution of modified Rakin Scale scores at 3-months in patients with and without target mismatch criteria. A modified Rankin scale score of 0 indicates no disability, 1 no clinically significant disability, 2 slight disability, 3 moderate disability but able to walk unassisted, 4 moderately severe disability, 5 severe disability, and 6 death.

**Figure 4.**
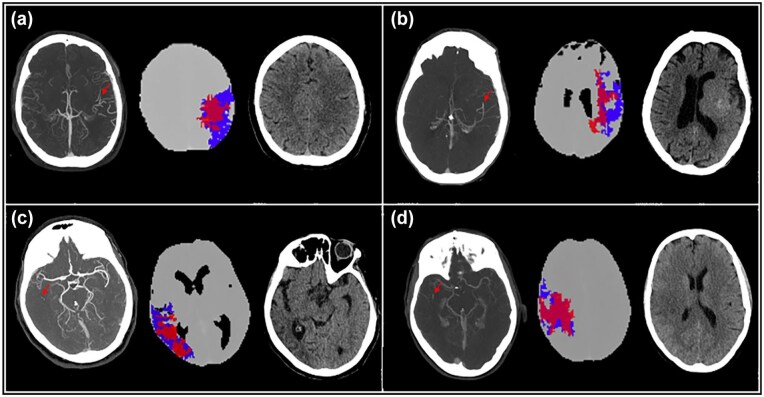
Illustrative cases of M2 DMVO patients with and without target mismatch criteria (mCTA, CTP penumbra map, NCCT at 24 hours; red arrow indicates the point of occlusion). Panel A: patient with target mismatch criteria (rCBF = 15 mL; penumbra = 43 mL; mismatch ratio = 3.8) and small final infarct volume (mRS at 3-months = 0). Panel B: patient without target mismatch criteria (rCBF = 27 mL; penumbra = 8 mL; mismatch ratio = 1.2) and small final infarct volume with hemorrhagic transformation (mRS at 3-months = 4). Panel C: patient without target mismatch criteria (rCBF = 35 mL; penumbra = 41 mL; mismatch ratio = 2.2) and large final infarct volume (mRS at 3-months = 5). Panel D: patient without target mismatch criteria (rCBF = 42 mL; penumbra = 18 mL; mismatch ratio = 1.4) and large final infarct volume (mRS at 3-months = 3). DMVO: distal-medium vessel occlusion; mCTA: multiphase CT-angiography; CTP: CT-perfusion; NCCT: non-contrast CT; rCBF: relative cerebral blood flow; mRS: modified Rankin scale.

The target mismatch criteria, defined as infarct core volume ⩽30 mL, penumbra volume ⩾10 mL, and mismatch ratio ⩾1.2, were more represented (*p* < 0.001) in patients with favorable than in those with unfavorable outcomes (47/68; 69.1% vs 14/47; 29.7%). In multivariable logistic regression analysis (Table 3), the target mismatch criteria were an independent predictor of functional independence (OR = odds ratio, 6.50 [2.37–17.77], *p* < 0.001), as well as NIHSS at admission (OR, 0.89 [0.82–0.96], *p* = 0.004) and pre-stroke mRS (OR, 0.42 [0.24–0.72], *p* = 0.002). Conversely, infarct core volume and successful recanalization were not proved to be independent predictors of good outcome. However, for recanalization there was a significant interaction between successful recanalization and target mismatch criteria on functional independence (OR, 3.82 [1.64–8.89], *p* = 0.002). The target mismatch criteria were also found to be an independent predictor of excellent clinical outcome (OR, 3.28 [1.30–8.31], *p* = 0.012) and final infarct volume (B, −35.52 [SE, standard error, 15.78], *p* = 0.004]) as shown in Table 4. As regarding safety outcomes, no differences in HT and sICH were noted between patients with and without target mismatch criteria. The mRS score distribution in patients with versus those without target mismatch criteria is shown in Figure 3. Figure 4 indicates an illustrative case of one DMVO patient with and three DMVO subjects without target mismatch criteria associated with good and poor functional outcome, respectively.

**Table 3.** Logistic regression analysis of predictors of favorable functional outcome (mRS 0–2 at 3-months).

	Univariable	*p*	Multivariable	*p*
	OR (95%CI)		OR (95%CI)	
Age	0.94 (0.90–0.98)	<0.001		
Sex	0.44 (0.19–1.02)	0.004		
Admission NIHSS	0.90 (0.84–0.96)	<0.001	0.89 (0.82–0.96)	0.004
mRS pre-stroke	0.49 (0.28–0.84)	<0.001	0.42 (0.24–0.72)	0.002
ASPECTS score	1.30 (0.91–1.85)	0.140		
Collateral Score	3.24 (1.37–7.64)	0.009		
rCBF Infarct core	0.94 (0.92–0.97)	0.004		
Target mismatch criteria	9.33 (3.71–23.48)	<0.001	6.50 (2.37–17.77)	<0.001
Onset-to-recanalization time	1.00 (1.00–1.00)	0.087		
mTICI score 2b-3	4.98 (1.66–14.91)	0.016		
Recanalization*target mismatch criteria			3.82 (1.64–8.89)	0.002

mRS: modified Rankin scale; OR: odds ratio; CI: confidence interval; NIHSS: National Institutes Health Stroke Scale; ASPECTS: Alberta Stroke Programme Early CT Score; rCBF: relative cerebral blood flow; mTICI: modified treatment in cerebral infarction score.

^*^Interaction term for recanalization and target mismatch criteria.

**Table 4.** Multivariable analysis of predictors of secondary outcomes.

Logistic regression	OR (95%CI)	*p* Value
mRS 0–1	3.28 (1.30–8.31)	0.012
sICH	0.98 (0.92–1.04)	0.084
Hemorrhagic transformation	1.99 (0.80–4.92)	0.135
Linear regression	B (SE)	
Final infarct volume	−35.52 (15.78)	0.004

mRS: modified Rankin scale; OR: odds ratio; CI: confidence interval; sICH: symptomatic intracranial hemorrhage; B: regression coefficient; SE: standard error.

## Discussion

In this study, we found that ⩽30 mL was the optimal threshold value for infarct core that predict favorable outcome in patients with M2 DMVO receiving EVT up to 24 h from stroke onset. This parameter was added to a prespecified favorable penumbra profile (penumbra volume ⩾10 mL and mismatch ratio ⩾ 1.2), previously associated with good outcome in this patient population,^11^ for generating target mismatch criteria applied in subjects with M2 occlusion. Both the infarct core volume ⩽30 mL and target mismatch criteria were more prevalent in patient with good than in those with poor outcome. More importantly, target mismatch criteria were independently associated with functional independence (mRS 0–2 at 3-months), whereas infarct core volume did not. These data expanded the results obtained by Sarraj et al.^11^ and were partially consistent with those emerging from a more recent study showing that, in M2 occluded patients treated with EVT, a favorable outcome was predicted by hypoperfused volume (infarct core plus penumbra volumes) and not by infarct core volume.^13^ Furthermore, our study was concordant with another recent publication^12^ in which infarct core volume was associated with outcome. This last investigation also described an association between recanalization and good outcome in M2 occluded patients undergoing EVT with infarct core volume ranging from 10 to 40 mL, penumbra volume >125 mL and hypoperfused volume >150 mL.^12^ These findings were consistent with our demonstration that good functional outcome after EVT was not directly related to successful recanalization, which is indeed effective only in patients with a favorable baseline imaging profile, including a small infarct core size. However, in contrast with Broocks et al.,^12^ we showed that patients with DMVO could achieve functional independence after EVT even in the presence of small penumbra volumes. In our study, nearly 30% of patients had a poor functional outcome despite target mismatch criteria at baseline and successful reperfusion. The underpinnings of this observation remain unclear, yet multiple factors might contribute to poor outcomes despite successful reperfusion and a favorable baseline perfusion profile, such as EVT-mediated complications, perfusion scotoma (i.e. ischemic core underestimated by CTP),^20^ no-reflow phenomenon,^21^ brain frailty,^22^ infarct location,^23^ and medical complications between post-EVT and 90-day. Taken together, these observations suggest that, as the treatment effect becomes significant only in those subjects meeting precise perfusion imaging parameters, the target mismatch proposed in this study could be an appropriate tool to select DMVO patients for treatment. In particular, we demonstrated that a small infarct volume was suitable for eligibility of patients with M2 occlusion for EVT. However, a post-hoc analysis of the MR-CLEAN LATE (Endovascular treatment vs no endovascular treatment after 6–24 h in patients with ischemic stroke and collateral flow on CT angiography),^24^ a trial that investigated EVT versus medical therapy in stroke patients presenting in the late window, found a direct interaction between penumbra volume extension and treatment effect in patients with M2 occlusion and a trend toward harmful reperfusion for patients with the smallest penumbra volume. Secondary analyses from DISTAL^7^ and ESCAPE-MeVO^8^ RCTs are warranted to elucidate whether perfusion imaging should help in selecting patients for EVT and which perfusion profiles should be applied. Nonetheless, our approach substantially differed from the baseline imaging selection criteria recently used in DISTAL^7^ and ESCAPE-MeVO^8^ RCTs, such as the evidence of a hypoperfusion-hypodensity mismatch (absence of hypodensity on the NCCT within ⩾90% of the area of the hypoperfused lesion on CTP)^7^ and a combination of visible hypoattenuation on NCCT plus good arterial collaterals and/or CTP penumbra mismatch^8^ respectively. In secondary analyses, the value of target mismatch as a predictor of outcome was confirmed by its independent association with an excellent outcome (mRS 0–1 at 3-months). Moreover, we found that target mismatch criteria were independently associated with final infarct volume (FIV) that was smaller in M2 occluded patients undergoing EVT with favorable than in those with unfavorable outcomes in univariable analysis, as previously reported.^25,26^ Intriguingly, in the study of Khan et al.^26^ the relationship between FIV and outcome became greater when FIV had a volume not exceeding 28 mL which seems in line with our infarct core volume threshold to define patients eligible for EVT. Surprisingly, we did not find a significant association between target mismatch criteria and safety outcomes. However, our results seem consistent with other studies^12,27^ not reporting a relationship between perfusion parameters and sICH. Moreover, distal MCA vessel occlusion, such as M3 and M4 segments not evaluated in our study, have been found to correlate with higher occurrence of sICH in comparison with M2 occlusions.^26^ In addition, the higher rates of good functional outcome in our study, compared to what has been observed in RCTs, might be explained by differences in the baseline imaging inclusion criteria. In fact, the automatic calculation of CT perfusion maps is currently considered a powerful tool to reduce imaging processing time and inter-operator variability, favoring reproducibility and rapid interpretation with better prognostic ability for good functional outcome over the qualitative CT perfusion mismatch approach^28,29^ used in those RCTs.^7,8^ Finally, we found that arterial collaterals were not independently associated with functional outcome. This finding is in line with previous studies showing that in patients with DMVO other measures of collateral blood flow, such as tissue-related collaterals,^30,31^ or venous outflow^32^ could play a more important role than pial arterial collaterals in maintaining the tissue at-risk of infarction viable over time following vessel occlusion. This study has some limitations. First, the small sample size collected at a single institution could weaken the consistency of our data. Second, as this study was based on a retrospective analysis, our findings require prospective validation. Third, we cannot exclude the influence of unmeasured confounders introducing potential selection bias due to the non-randomized design of our study. Fourth, this study did not include patients with anterior or posterior cerebral artery occlusions, mainly because of their lower frequency and the uncertainty regarding the significance of target mismatch criteria in these cases.^33^ Fifth, the definition of an M2 branch to be dominant, non- or codominant is subjective per se and often not clearly assessable due to the complex and variable anatomy with inherently inconsistent anatomic terminology,^12^ which could limit our findings. Sixth, as recommended by the vendor, admission infarct core volume was calculated using rCBF < 40% as threshold value instead of rCBF < 30% which is the widely adopted reference threshold value. CTP pitfalls in estimating the ischemic core, such as perfusion scotoma and ghost core as well as infarct growth beyond recanalization, might have influenced the relationship between baseline ischemic core and outcomes in our study. However, 24-h infarct volumes were similar to baseline CTP-estimated core volumes in our sample, likely mitigating the effect of this potential limitation.^34^ Finally, a single CTP software was used, and confirmation of our findings using other platforms is needed.

## Conclusions

We demonstrated that the benefit of EVT in patients with DMVO, particularly those with M2 non- or codominant occlusions, was associated with favorable baseline CTP parameters, collectively named target mismatch criteria. While recent trials focusing on large core have questioned the necessity of imaging selection for EVT, our data indicate that perfusion imaging might become crucial for informing EVT decision-making in the DMVO population.
